# Gloves in Operative Surgery

**Published:** 1902-12-27

**Authors:** 


### GLOVES IN OPERATIVE SURGERY.

At a recent meeting of the New York Academy of Medicine, reported in the New York *Medical Record*, Dr. G. R. Fowler read a paper on the "Pre-operative Preparation of the Surgeon and his Assistants," which elicited various opinions on the use of gloves in surgery. The crux of the question seems to hinge upon the fact that although immediately after disinfection, say by alcohol, the hands may be sterile enough, microbes soon come to the surface again when the hands are placed in warm water. As for rubber gloves, he said that they were useful, but that their utility was limited to certain examinations in very septic cases, and that they could not be employed where delicate tactile sense was required. There was scarcely an operation in surgery that did not risk the tearing or the puncture of the gloves, while the interference with the tactile sense and the catching of the needle in the glove made them very inconvenient. Worst of all, however, was the fact that the poulticing of the hands which arose from the use of the rubber gloves caused the passage of myriads of micro-organisms to the surface of the skin, so that when the gloves were torn or punctured infection was actually favoured by their use. Dr. Ware said that the objection to the use of rubber gloves which was founded on their producing sweating was met by the substitution of cotton gloves, under which, as had been shown by experiment, the hands were more nearly sterile than when under rubber. Dr. Joseph Wiener, jun., thought that, notwithstanding the objections which had been raised to the use of rubber gloves, they had their advantages. By perseverance in their use most of the difficulties first experienced had vanished. He would not think of doing any abdominal operation without rubber gloves; he did not find that he punctured them more than about once in 15 operations; and he could palpate with the gloves on just as well as without them. Lately it had been found at the hospital that it was a great improvement to make use of dry sterile gloves well powdered with sterile talcum powder. Although he ordinarily perspired freely, he had been able by means of the talcum to do an operation and keep the skin under the glove dry the whole time. Many interesting points were raised in the course of the discussion; for example, the necessity of covering the head—and if the surgeon insisted on wearing a beard, the beard also—was pointed out. It was also shown that the brow should be protected by several thicknesses of gauze bandage to prevent the falling of septic perspiration into the wound, a disaster which not infrequently occurred.

